# Value of Perampanel as Adjunctive Treatment for Partial-Onset Seizures in Epilepsy: Cost-Effectiveness and Budget Impact Analysis

**DOI:** 10.3389/fpubh.2021.670108

**Published:** 2021-07-06

**Authors:** Donger Zhang, Xia Li, Jing Ding, Xiatong Ke, Wenpei Ding, Yinan Ren, He Xu, Hongchao Li, Aixia Ma, Wenxi Tang

**Affiliations:** ^1^School of International Pharmaceutical Business, China Pharmaceutical University, Nanjing, China; ^2^Center for Pharmacoeconomics and Outcomes Research, China Pharmaceutical University, Nanjing, China; ^3^Department of Neurology, Zhongshan Hospital, Fudan University, Shanghai, China

**Keywords:** value, perampanel, cost-effectiveness, budget impact, epilepsy, partial-onset seizures

## Abstract

**Introduction:** China has ~6 million patients with active epilepsy every year, around 60% of whom suffer from partial-onset seizures. Perampanel (PER) is a novel anti-epileptic drug for partial-onset seizures. PER has been included in the latest Chinese National Reimbursement Drug List (NRDL) in 2020. However, there is still a lack of evaluation evidence on the value of PER in China.

**Methods:** This study selected a health system perspective. A Markov model was established to simulate the lifelong transition of different response levels and calculate the number of seizures in Chinese patients. Based on the utility value and mortality risk, the life years and quality-adjusted life years (QALYs) of patients using PER vs. lacosamide (LCM) were estimated. Efficacy data were derived from clinical trials and the literature. Cost data (in US dollars) included drug costs and medical service costs. A lifetime horizon was adopted. Health outcomes and costs were discounted at an annual discount rate of 5%. Deterministic sensitivity analysis, probability sensitivity analysis, and scenario analysis were performed. The impact of the inclusion of PER in the NRDL on the medical insurance budget over 3 years (2021–2023) was also estimated.

**Results:** Cost-effectiveness analysis indicates that 8 mg/day of PER increases QALYs by 0.054 and saves costs by $2,390 compared with 400 mg/day of LCM. 4 mg/day of PER increases QALYs by 0.010 and saves costs by $860 compared with 200 mg/day of LCM. Deterministic sensitivity analysis reveals that utility value and the extreme discount rate are the factors with the greatest impact on the incremental cost-effectiveness ratio. Probabilistic sensitivity analysis and scenario analysis show that the results are robust. Budget impact analysis indicates that after inclusion of PER in the NRDL, the incremental budget would be $1.28, $2.83, and $4.56 million from 2021 to 2023, respectively, but covering more eligible epileptic patients in the same time (1,918, 4,287, and 8,983, respectively).

**Conclusion:** PER (8 or 4 mg/day) is of relatively high value as an add-on therapeutic regimen for partial-onset seizures in China because of its dominate advantage of cost-effectiveness over LCM and acceptable budget impact.

## Introduction

Epilepsy is a chronic non-communicable disease of the brain that affects people of all ages. There are ~50 million patients with epilepsy worldwide, making it one of the most common neurological diseases in the world (1). Nearly 80% of epileptic patients live in low- and middle-income countries. China has ~6 million patients with active epilepsy (AE, defined as two or more unprovoked seizures in the past year) every year, and 60% of them suffer from partial-onset seizures (2, 3) with a mortality risk of 2–3 times that of the general population (4).

Epilepsy accounts for 5% of the global economic burden of mental illnesses (5). Long-term administration of anti-epileptic drugs (AEDs) and other costs of diagnosis and treatment impose a heavy economic burden to families. In 2009, a study conducted in Southwest China showed that the average annual cost (in US dollars) of epileptic patients was $773, in which the cost of AEDs amounted to $243 (6). In 2015, another study on the disease burden in central China showed that the average annual cost of epileptic patients reached $949 (7). Moreover, the stigma of epilepsy brings about a serious psychological burden to patients and their families, which discourages the patients from seeking treatment and reduces their quality of life (1). As for children and adolescents, suffering from epilepsy imposes a lifelong impact on their social, emotional, and occupational development (8). Therefore, the prevention and treatment of epilepsy is not only a medical problem, but also a vital public health and social problem.

Epilepsy is a treatable disease, with AEDs being the preferred therapeutic option and the most important treatment approach. Up to 70% of epileptic patients achieve seizure remission for a relatively long time through proper diagnosis and standardized use of AEDs (9). However, in developing countries, most epileptic patients cannot receive reasonable and effective treatment. In China, the treatment gap for AE patients is ~60% (3, 10). Epileptic patients therefore have significant unmet medical needs.

As recommended drugs for the treatment of refractory focal epilepsy in adults, third-generation AEDs including perampanel (PER) and lacosamide (LCM) show advantages in terms of efficacy, safety, and tolerability over first- and second-generation AEDs (11). PER (Fycompa®, Eisai Co., Ltd., Tokyo, Japan) is a first-in-class non-competitive highly selective α-amino-3-hydroxy-5-methyl-4-isoxazole propionic acid receptor antagonist. Several multi-center, randomized-controlled, phase III clinical trials have shown that compared with placebo, PER (4–12 mg/day) considerably reduces seizure frequency and improves response rate with acceptable tolerability in epileptic patients (12–15). LCM is one of the sodium channel blockers in AEDs. Two phase III clinical trials have shown that compared with placebo, LCM substantially reduces seizure frequency with good tolerability (16, 17). In the 2018 guidelines from the American Academy of Neurology and the American Epilepsy Association (AAN/AES), the grades of evidence-based recommendation for PER and LCM are A and B, respectively (11). Cost-effectiveness (CE) evidence and budget impact analysis (BIA) of PER have been reported in other countries (18, 19). Some studies have also explored the CE of LCM as an add-on to conventional AED therapy (20, 21). All these studies have demonstrated that PER or LCM as an add-on therapy for uncontrolled or refractory epilepsy is a cost-effective regimen (18, 20, 21).

In 2019, China approved PER for add-on therapy in adults and children over 12 years of age with partial-onset seizures (with or without secondary generalized seizures). In December 2020, PER was included in China's latest National Reimbursement Drug List (NRDL; LCM was released to the market in 2018 and included in the NRDL in 2019). Due to the limitations of the public budget, economic efficiency has become one of the most important factors for inclusion in the NRDL of China since 2018.

The economic efficiency of LCM compared with conventional therapy was proven in 2010. Bolin et al. showed that LCM was cost-effective as an add-on therapy for uncontrolled partial-onset seizures (21). In 2018, the economic efficiency of PER as an add-on therapy for primary systemic tonic-clonic seizures was confirmed in Spain (18). However, the economic efficiency of PER therapy for partial-onset seizures in China remains unknown, and there is a lack of comparison between PER and LCM. From the perspective of resource allocation, even if the drugs are economical, whether they can be afforded by public funds is still the threshold for inclusion. According to the Institute for Clinical and Economic Review (ICER) Value Framework 2.0, the economic efficiency and affordability of a drug should be simultaneously included in its value evidence (22). Since 2019, the evidence from CE analysis (CEA) and BIA has also become economic evidence that is compulsory to be submitted for inclusion in the NRDL of China.

The aim of this study is to evaluate the value of PER as an add-on regimen to the treatment of partial-onset seizures in China and provide evidence on both its CE and affordability.

## Materials and Methods

### Model Structure

As epilepsy is a lifetime chronic disease, we used the Markov model to evaluate lifelong effectiveness and costs of PER + AEDs vs. LCM + AEDs in patients with partial-onset seizures in China. The health state was classified according to seizure frequency, namely: ≥53 times/year, 13–52 times/year, 1–12 times/year, and no seizures. The seizure frequency was dependent on the response level after medication (the scale of the decrease in baseline number of seizures). More specifically, distribution of response levels over each cycle period were mapped to health states defined by seizure frequency. The mapping calculation was based on clinical trials. There are six response levels: seizure-free, response rate of 75–99%, response rate of 50–74%, response rate <50%, increased seizures, and maintenance therapy (including patients withdrawn from the trial due to adverse reaction or other reasons). The cycle period of the model was set to 4 months (consistent with the medication regimen). This model adopted a lifetime horizon, and the cycle of the model was terminated when the proportion of dead patients reached 99.9%. According to latest guidelines for pharmacoeconomic evaluation in China (23), health outputs and costs were discounted at an annual discount rate of 5%.

In particular, two dose comparison groups were set up in this study, namely PER 8 mg/day vs. LCM 400 mg/day and PER 4 mg/day vs. LCM 200 mg/day, as adjunctive treatment. This design was based on comprehensive consideration of the defined daily dose (DDD) recommended by the World Health Organization (WHO) (24), the availability of clinical trial data (12, 16), and the actual clinical dose used in China indicated by clinicians (25).

**Figure 1:**
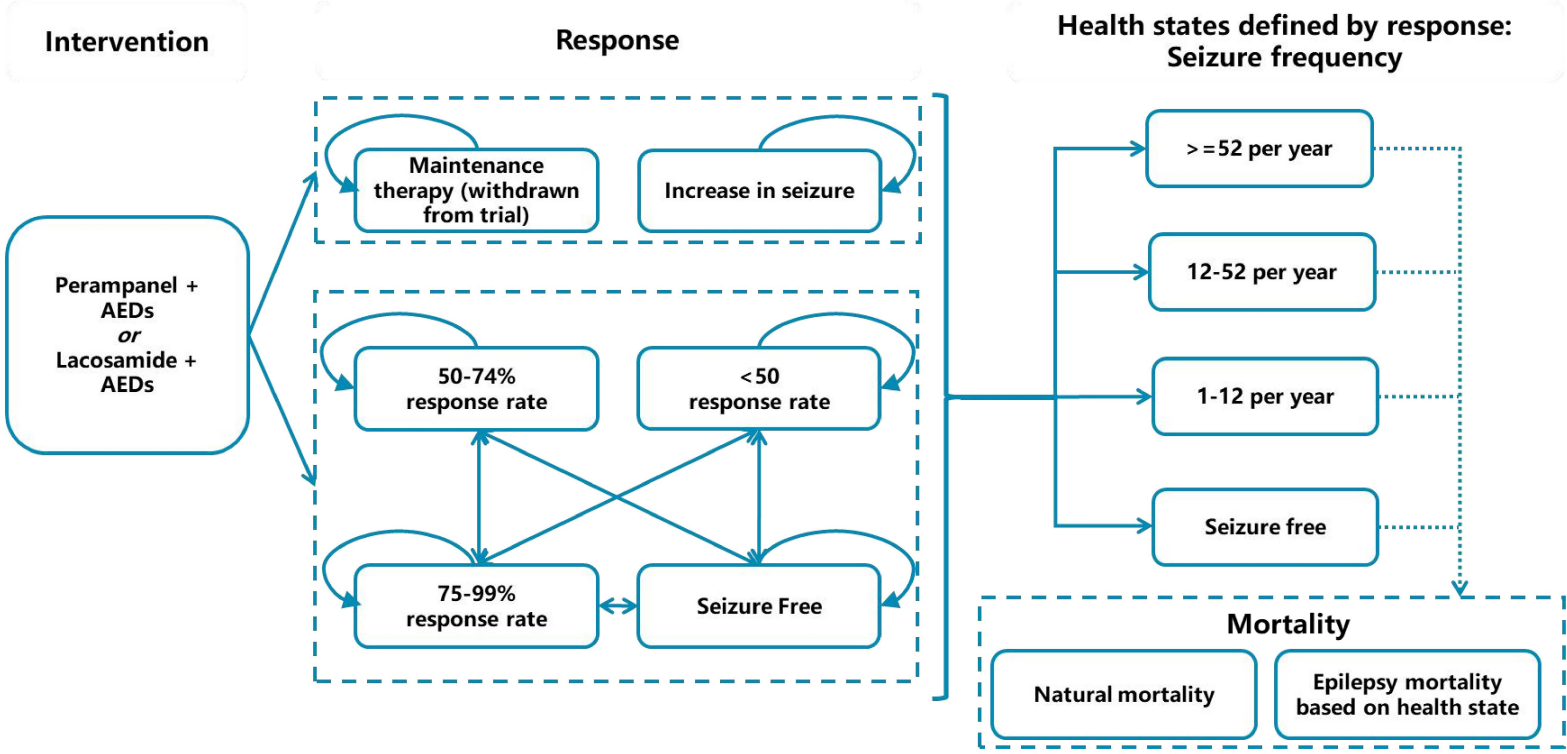
Model structure. AED, antiepileptic drug.

The model structure is shown in Figure 1.

### Health Outcomes and Data Sources

Health outcomes included the number of epileptic seizures, life years (LYs), and quality-adjusted life years (QALYs).

For the first cycle in the model, response rates were derived from clinical trials 335 (trial registration number: NCT01618695, sample size: 704) and EP0008 (trial registration number: NCT01710657, sample size: 548), both of which include reasonably large sample sizes in Chinese populations (199 Chinese in trial 335, 406 Chinese in trial EP0008). The baseline seizure distribution of patients by health state was displayed in Table 1, as LCM 4 mg/day and LCM 8 mg/day was assumed to have the same baseline seizure frequency with PER 4 mg/day and PER 8 mg/day, respectively. Other baseline information of patients involved in the clinical trials is listed in Supplementary Table 1. Because the two trials were not head-to-head trials, we used an indirect comparison method in this study and acquired the risk ratio values of PER and LCM response rates with conventional therapy as the bridge. Because LCM lacked data on the proportion of patients with increased epileptic seizures, we assumed the proportion of patients whose seizures increased by <25% to be equal to that of the PER group. We obtained the proportion of patients whose seizures increased by ≥25% from the data reported in another clinical trial of LCM (26). The initial distributions of response rates are detailed in Table 2.

**Table 1:** Baseline seizure distribution in Markov model, %.

	**≥53 times/year**	**13–52 times/year**	**1–12 times/year**	**Seizures free**
PER 8 mg/day (*N* = 48)	87.76	12.24	0	0
LCM 400 mg/day (*N* = 133)	87.76	12.24	0	0
PER 4 mg/day (*N* = 43)	86.67	13.33	0	0
LCM 200 mg/day (*N* = 135)	86.67	13.33	0	0

*PER, perampanel; LCM, lacosamide*.

**Table 2:** Response distribution in the first cycle, %.

	**Maintenance therapy**	**Increase in seizure**	** <50% Response**	**50–74% Response**	**75–99% Response**	**Seizure free**
PER 8 mg/day (*N* = 48)	9.43	26.42	20.75	22.64	13.21	7.55
LCM 400 mg/day (*N* = 133)	16.33	28.17	0.92	33.64	17.62	3.31
PER 4 mg/day (*N* = 43)	6.52	28.26	41.30	4.35	13.04	6.52
LCM 200 mg/day (*N* = 135)	8.64	22.20	20.38	26.43	18.49	3.85

*PER, perampanel; LCM, lacosamide*.

From the second cycle, transition probabilities of response rate were obtained from long-term follow-up data (27). The model converted 5-year transition probabilities according to the following equation (28):

$$\begin{array}{l} {\text{P}}_{4\text{month}}=1-\text{exp}\left[{\left(1/15\right)}^{*}\;\left[\text{ln}\left(1-{\text{P}}_{5\text{year}}\right)\right]\right] \end{array}$$

where P_5year_ denotes the 5-year probability, P_4month_ denotes the 4-month probability, and 4 months accounts for 1/15 of 5 years. Neligan et al. only reported the probability of response rates transitioning from <50 to 50–99% (27). Here, we allocated the transition of response rates from <50 to 50–74% or 75–99% according to the proportion of patients corresponding to the response rates of 50–74% and 75–99% in the trial EP0008 (16). The transition probabilities of response rates are listed in Supplementary Table 2.

Natural mortalities were obtained from the life table of natural mortalities in China published by the WHO (29). The seizure-free population was considered to have the same mortality risk as the general population. Mortality risks for different seizure frequencies were based on the data reported by Nilsson et al. (30), and the RR values are listed in Table 3.

**Table 3:** Inputs in the CE model.

	**Base case**	**DSA Range^**a**^**	**Distribution**	**Source**
**Relative risks of mortality**				
≥53 seizures/year	10.16	2.94–35.18	–	(30)
13–52 seizures/year	8.64	2.88–25.93	–	(30)
≤ 12 seizures/year	7.21	2.52–20.6	–	(30)
**Drug costs per 4 months ($)**				
PER 4 mg/day	878	±10%	Gamma	MENET, 335 clinical trial
AEDs – PER 4 mg/day group	692	−20%−0%	Gamma	
PER 8 mg/day	1,754	±10%	Gamma	MENET, 335 clinical trial
AEDs – PER 8 mg/day group	827	−20–0%	Gamma	
LCM 200 mg/day	1,484	−20–0%	Gamma	MENET, (16)
AEDs – LCM 200 mg/day group	695	−20–0%	Gamma	
LCM 400 mg/day	2,968	−20–0%	Gamma	MENET, (16)
AEDs – LCM 400 mg/day group	549	−20–0%	Gamma	
**Medical costs per 4 months ($)**				
≥53 seizures/year	571	±20%	Gamma	Health care documents^b^, KOL
13–52 seizures/year	441	±20%	Gamma	Health care documents, KOL
≤ 12 seizures/year	273	±20%	Gamma	Health care documents, KOL
Seizure free	180	±20%	Gamma	Health care documents, KOL
**Health Utilities per 4 months**				
≥53 seizures/year	0.619	±0.15	Beta	(31)
13–52 seizures/year	0.628	±0.12	Beta	(31)
≤ 12 seizures/year	0.673	±0.14	Beta	(31)
Seizure free	0.711	±0.14	Beta	(31)

a *To echo the medical pricing reform in China, we assumed the drug prices could only decrease and the service item prices increase. In addition, we assumed a narrower range (±10%) of Perampanel price according to the lowest price provided by Eisai Co., Ltd.*

b *The health care documents from the 9 provinces medical security bureaus.*

*CE, cost-effectiveness; DSA, deterministic sensitivity analysis; PER, perampanel; LCM, lacosamide; AED, antiepileptic drug*.

### Costs

From a health system perspective, only direct medical costs were taken into account in this study, including drug costs and medical service costs (covering outpatient, emergency, and inpatient treatment costs). For drug costs, the unit price of drugs was multiplied by their daily dose to obtain the unit cost. AED costs were derived from the unit price multiplied by the use ratio of various drugs in clinical trials (335, EP0008). The unit price of drugs was derived from the median national price in the China Drug Bidding Database (shuju.menet.com.cn). In all cases, it was included as the price of the largest sales specification and converted into the unit price of the smallest dose. Medical service costs were derived from the unit price multiplied by the frequency of medical visits, and details are displayed in Supplementary Table 3. Because of the lack of data on medical visits for epilepsy, the items of medical services and the frequency of medical visits were acquired from the opinions of 18 clinicians from different hospitals in China. The unit price of medical services was derived from the median of documented data in the medical insurance bureaus of nine provinces and cities in China. Because the service prices were inconsistent among different levels of hospitals, we adjusted the service prices according to the proportion of medical visits to second- and third-level medical institutions (32).

All costs used in this study were converted from Chinese yuan to US dollars [1 dollar = 6.5408 yuan (33)]. Details are provided in Table 3.

### Health Utilities

Health state utility values were acquired from the National Health and Wellness Survey of Kantar Health (31). Details are summarized in Table 3.

### Base-Case Analysis

Costs were calculated by simultaneously taking into account the usage of all drugs (including PER, LCM, and AEDs) and medical service resources by the patients in different states. Finally, the number of seizures, LYs, QALYs, and incremental CE ratio (ICER) related to PER + AEDs vs. LCM + AEDs were obtained.

### Uncertainty Analysis

We performed one-way deterministic sensitivity analysis (DSA) using the 95% confidence interval reported in the literature as the variation range. The discount rate ranged from 0 to 8% (23). The impact of the extreme discount rate (effectiveness: 8%, cost: 0%; effectiveness: 0%, cost: 8%) on the results was taken into account. Assuming that the distribution proportion of PER entering maintenance therapy, response rate of 75–99%, and seizure-free state fluctuates by 20% for DSA. The variation range of other parameters that lacked documented reports was assumed based on KOL (Key Opinion Leader) opinions.

Probabilistic sensitivity analysis (PSA) was performed using Monte Carlo simulation with 10,000 iterations. The standard deviation of utility values was derived from the literature (31). The standard deviation of costs was obtained from the standard deviation of AED prices and the distribution range of KOL opinions. Parameter distributions came from classical assumption (28). The parameter characteristics of mortality, cost, and utility value are listed in Table 3. In addition to the parameters listed in Table 3, we assumed that LY conforms to a normal distribution, with its mean and standard deviation derived from the model.

In basic analysis, we assumed that there was no transition from <50% response rate to increase in seizure. However, we found this assumption was uncertain compared with other studies. Considering that the transition patterns of response rates are the basis of the cycle in the model, we designed a scenario analysis according to the pharmacoeconomic study of Tremblay et al. (18): assuming that there are probabilities for patients with a response rate of <50% transitioning to the state of increased seizures, and the transition is expressed by the following equation:

*P* <50% to increase in seizures = *P* increase in seizures ^*^ (1 – P responsive).

### Budget Impact

**Figure 2:**
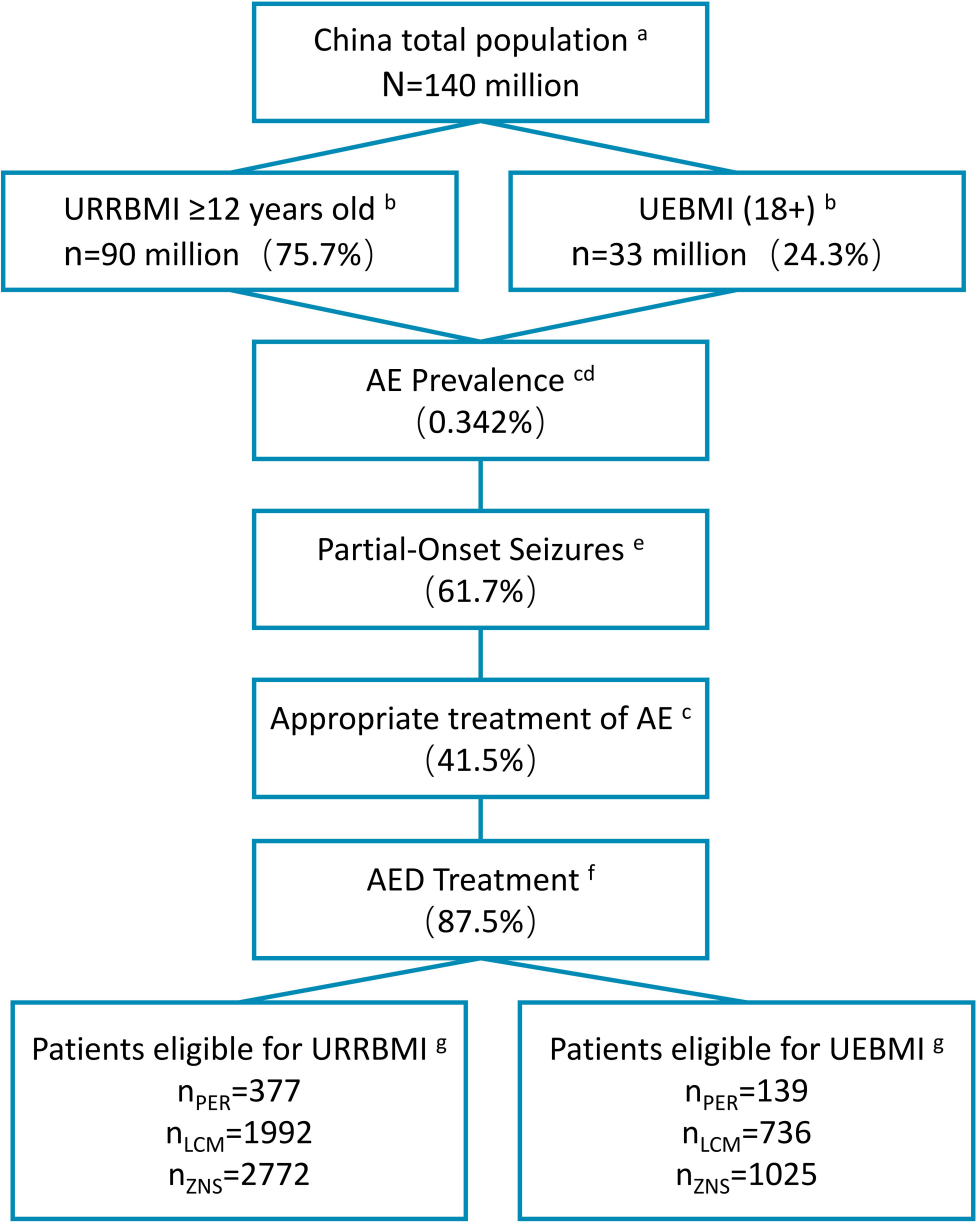
Patient disposition in BI model (2020). ^a^Data from the National Bureau of Statistics of China (data.stats.gov.cn). ^b^Basic medical insurance participation data from reports of medical insurance bureau (nhsa.gov.cn), and proportion of people over 12 years old from 2010 Census Report (data.stats.gov.cn). ^c^Prevalence of active epilepsy and rate of standardized treatment from Ding et al. (10). ^d^Annual growth rate of the prevalence during 2021–2023 from Song et al. (34) ^e^Proportion of patients with partial seizures from Yu et al. (2). ^f^Standardized treatment rate of drugs from investigation results of 18 clinical experts. ^g^Market shares of three drugs provided by Eisai Co., Ltd. (Table 4). BI, budget impact; URRBMI, Urban Rural Resident Basic Medical Insurance; UEBMI, Urban Employee Basic Medical Insurance; PER, perampanel; LCM, lacosamide; ZNS, zonisamide.

We analyzed the impact of the inclusion of PER in the NRDL on the medical insurance budget in 2021–2023. The target population comprised participants in basic medical insurance aged ≥12 years with partial-onset seizures and undergoing standardized drug treatment (Figure 2). Taking into account the substitution of other similar drugs by PER, we added zonisamide (ZNS) as a reference drug in addition to LCM. The market share of the three drugs in 2020–2023 was obtained by Eisai Co., Ltd. based on the actual market situation (Table 4). Cost data were derived from the CEA results, and the distribution proportion of patient health states before and after treatment is summarized in Supplementary Table 4. For the two groups of daily doses, we obtained the use ratio of clinical patients for different daily doses of PER and LCM by consulting KOL, and this set of data was used for weighting in the cost calculation. The medical insurance reimbursement policy is provided in Supplementary Table 5. DSA was performed on epidemiological parameters, use ratio of clinical doses, and cost parameters that may affect budget impact (BI). The distribution ranges of the parameters are given in Table 5.

**Table 4:** Market share (Before and after the inclusion of PER in the NRDL), %.

**Drugs**		**Before**			**After**		
	**2020**	**2021**	**2022**	**2023**	**2021**	**2022**	**2023**
PER	0.06	0.14	0.24	0.34	0.35	0.7	1.25
LCM	0.32	0.66	1.15	1.61	0.62	1.09	1.42
ZNS	0.48	0.41	0.34	0.29	0.39	0.31	0.24
Total	0.86	1.21	1.73	2.24	1.36	2.1	2.91

*Market shares are derived using the overall market of oral anti-epileptic products as the prediction baseline and the data of PER in the baseline year (2020) as the data in Q1 of that year. The impact of ± 10% variation in the annual market share data of various drugs on the BI results after inclusion of PER in the NRDL is observed in DSA.*

*NRDL, National Reimburesement Drug List; PER, perampanel; LCM, lacosamide; ZNS, zonisamide*.

**Table 5:** Inputs in the BI model.

**Parameters**	**Base case**	**DSA range**	**Source**
**Epidemiology**			
AE prevalence, 2013 (%)	0.24	0.22–0.33	(10, 35, 36)
Annual growth rate of prevalence(%)	5.20	±30%	(34)
**Proportion of clinical dose used by patients**^**a**^ **(%)**			
Proportion of patients taking PER 4 mg/day	60.00	50.00–80.00	KOL
Proportion of patients taking LCM 200 mg/day	68.42	50.00–80.00	KOL
**Drug costs**^**b**^			
Annual cost of PER	1,211	±10%	MENET
Annual cost of AEDs-PER	762	−30–0%	MENET, 335 clinical trial
Annual cost of LCM	1,924	−30–0%	MENET, (16)
Annual cost of AEDs-LCM	592	−30–0%	MENET, (16)
Annual cost of ZNS	700	−30–0%	MENET
Annual cost of AEDs-ZNS^c^	670	−30–0%	MENET
Annual cost of AEDs	710	−30–0%	MENET, 335 clinical trial
**Direct medical costs**^**d**^	–	–	–

a *Assuming that the proportion of other AEDs administered by the patients taking ZNS is the average of PER and LCM, because ZNS lacks available clinical trial data.*

b *The sum of the use ratio of PER 8 and 4 mg/day is 100%, which is similar to LCM. These data are derived from opinions of 18 KOLs.*

c *Drug costs are weighted according to KOL opinions.*

d *Medical service costs in the BI model are exactly the same as used in the CE model (omitted here), except for the parameter range of ±30% in DSA.*

*BI, budget impact; DSA, deterministic sensitivity analysis; AE, active epilepsy; PER, perampanel; LCM, lacosamide; ZNS, zonisamide; AED, antiepileptic drug*.

## Results

### Base-Case Analysis

The results of base-case analysis (Table 6) show that PER has an dominate advantage over LCM in terms of CE. Compared with LCM 400 mg/day, PER 8 mg/day reduces the number of seizures per capita by 141 times, with an incremental LY of 0.061, an incremental QALY of 0.054, and a direct medical cost saving of $2,390. Compared with LCM200 mg/day, PER 4 mg/day reduces the number of seizures per capita by 72 times, with an incremental LY of 0.012, an incremental QALY of 0.010, and a cost savings of $860.

**Table 6:** Base case analysis results.

	**Absolute**		**Incremental**		**Absolute**		**Incremental**	
	**PER 8 mg/day**	**LCM 400 mg/day**		**Change%**	**PER 4 mg/day**	**LCM 200 mg/day**		**Change%**
Seizures	467	608	−141	−23.22	717	789	−72	−9.14
LYs	4.940	4.879	0.061	1.26	5.17	5.158	0.012	0.24
QALYs	3.137	3.083	0.054	1.75	3.278	3.268	0.010	0.31
Drug costs	9,144	11,462	−23,18	−20.22	7,304	8,229	−925	−11.24
Medical costs	7,001	7,073	−72	−1.01	7,400	7,335	65	0.89
Total costs	16,145	18,535	−2,390	−12.89	14,704	15,564	−860	−5.52

*PER, perampanel; LCM, lacosamide; LY, life year; QALY, quality-adjusted life years*.

### Uncertainty Analysis

#### Deterministic Sensitivity Analysis

**Figure 3:**
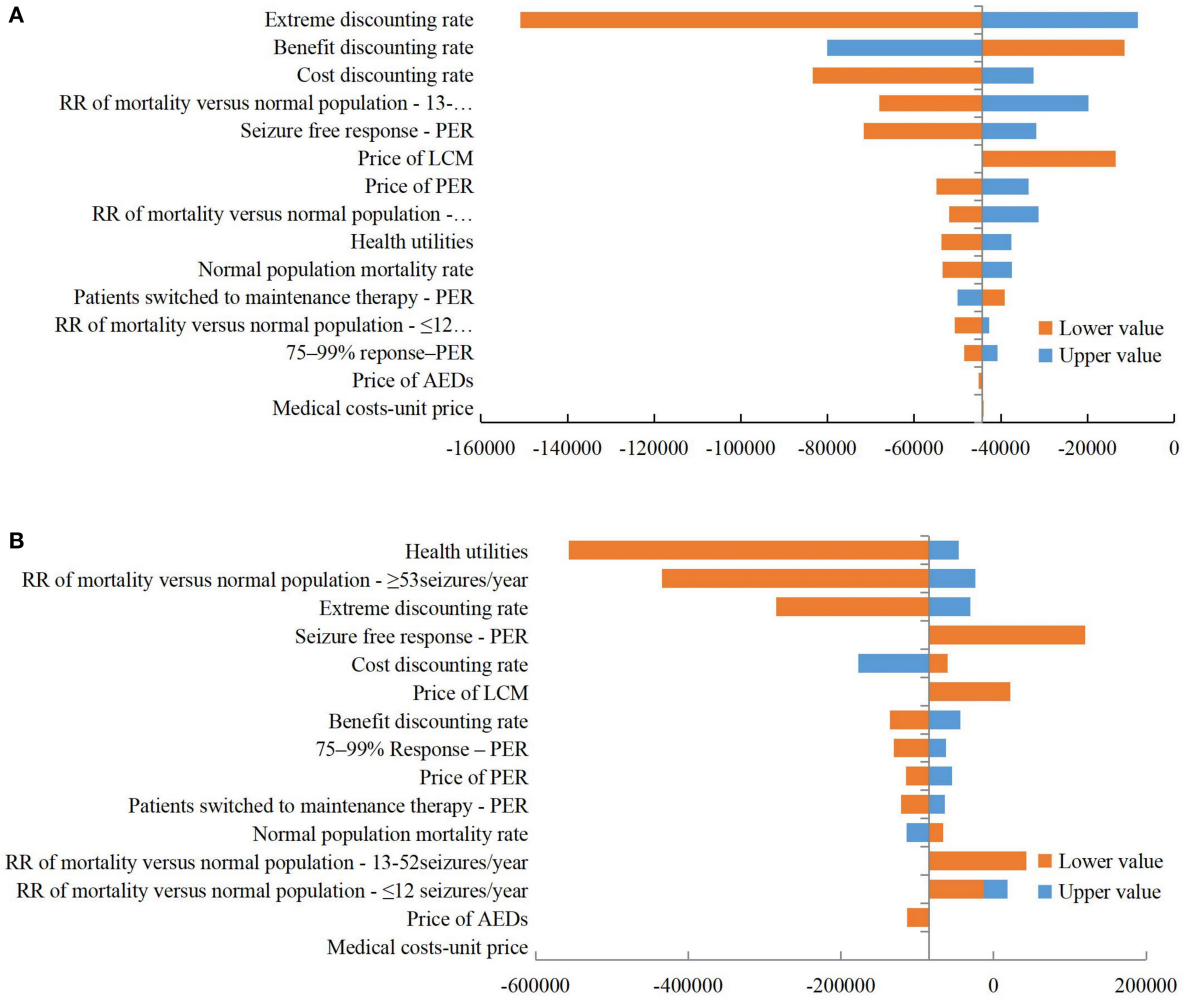
Tornado diagram of deterministic sensitivity analysis. **(A)** PER 8 mg/day vs. LCM 400 mg/day; **(B)** PER 4 mg/day vs. LCM 200 mg/day. ER, perampanel; LCM, lacosamide; RR, relative risk; AED, antiepileptic drug.

The DSA results (Figure 3) show that the ICER of PER 8 mg/day vs. LCM 400 mg/day group ranges from $150,911 to $8,418/QALY, with the extreme discount rate having the greatest impact on ICER. The ICER of PER 4 mg/day vs. LCM 200 mg/day group ranges from $556,653 to $119,970/QALY, with the utility value having the greatest impact on ICER. The price of AEDs and the unit price of medical services have little impact on ICER in both groups.

#### Probabilistic Sensitivity Analysis

**Figure 4:**
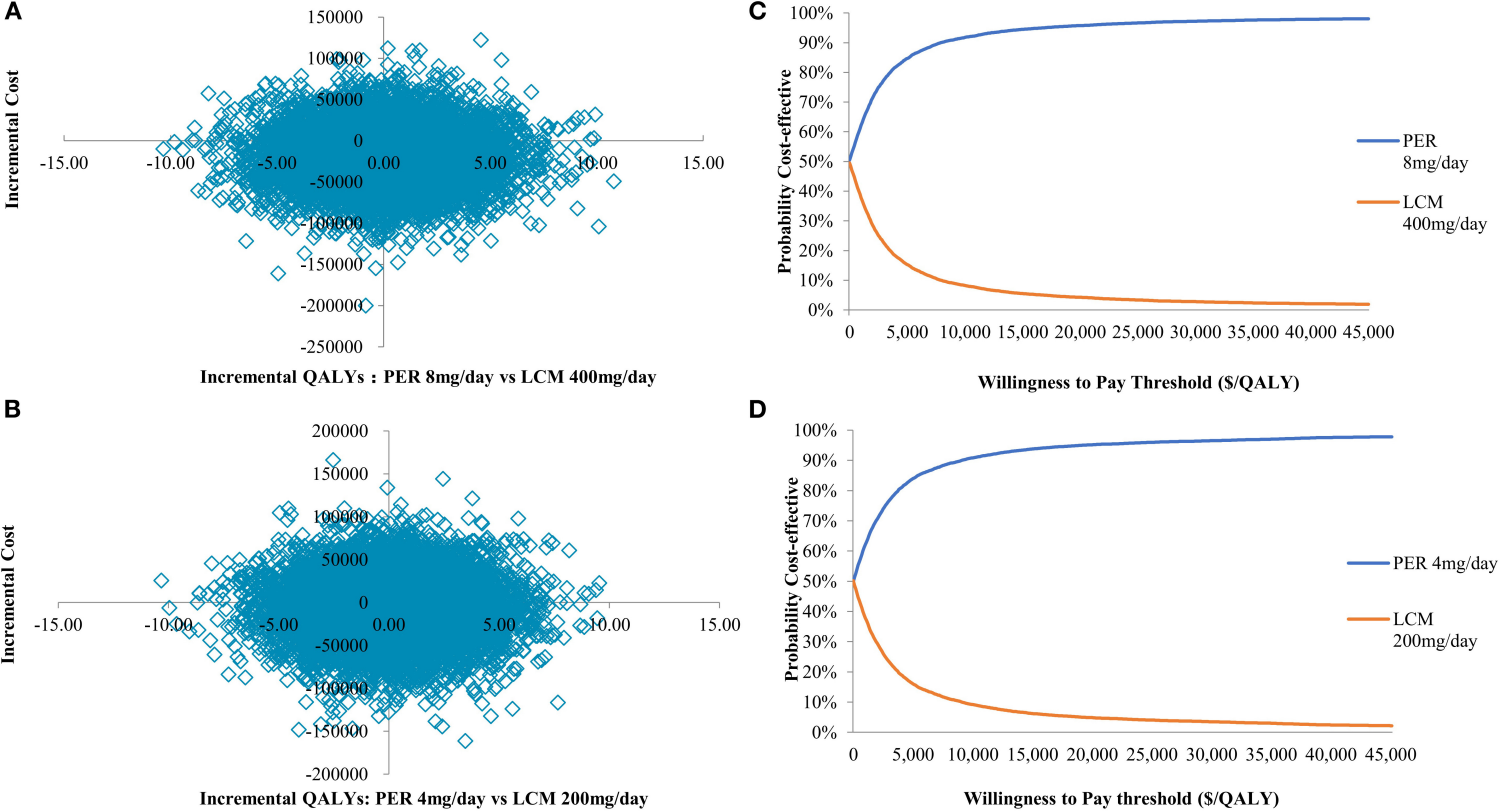
**(A,B)** Scatterplot of CE plane; **(C,D)** Cost-effectiveness acceptability curve. PER, perampanel; LCM, lacosamide.

The PSA results (Figure 4) show that the different dose groups of PER have a large probability of being economical at various levels of willingness-to-pay. The one-to-three times gross domestic product (GDP) per capita was $10,838–$32,515 in China in 2019 (data.stats.gov.cn).

#### Scenario Analysis

The results of scenario analysis (Table 7) show that compared with LCM 400 mg/day, PER 8 mg/day achieves an incremental QALY of 0.039 and a cost saving of $2,747, which is still the regimen with dominate advantage. However, compared with PER 4 mg/day, LCM 200 mg/day results in an incremental QALY of 0.005 and an incremental cost of $554. The ICER is $105,194/QALY, higher than three times GDP per capita [$32,515], indicating that LCM is not cost-effective. This result may be attributed to the relatively small incremental QALY, which may not meet the minimal clinically important difference.

**Table 7:** Scenario analysis results.

	**Absolute**		**Incremental**		**Absolute**		**Incremental**	
	**PER 8 mg/day**	**LCM 400 mg/day**		**Change%**	**PER 4 mg/day**	**LCM 200 mg/day**		**Change%**
Seizures	530	633	−103	−16.23	854	873	−19	−2.16
LYs	4.912	4.868	0.044	0.90	5.122	5.127	−0.005	−0.09
QALYs	3.111	3.072	0.039	1.24	3.233	3.238	−0.005	−0.16
Drug costs	7,198	9,947	−2,749	−27.63	5,800	6,496	−696	−10.71
Medical costs	7,137	7,135	2	0.03	7,649	7,507	142	1.89
Total costs	14,335	17,082	−2,747	−16.08	13,449	1,4003	−554	−3.95
					ICER per seizure avoided ($/seizure)	29.41
					ICER per LY ($/year)	116,275.56		
					ICER per QALY ($/QALY)	105,193.94		

*PER, perampanel; LCM, lacosamide; LY, life year; QALY, quality-adjusted life years; ICER, incremental cost-effectiveness ratio*.

### Budget Impact Analysis

#### Base-Case Analysis

After the inclusion of PER in the NRDL, it is expected that the target population using PER will increase by 1,918; 4,287; and 8,983 in 2021–2023, respectively, with a total population increase from 6,557 to 21,774. In contrast, the target populations of LCM and ZNS will decrease by a total of 3,531 in the 3 years.

**Figure 5:**
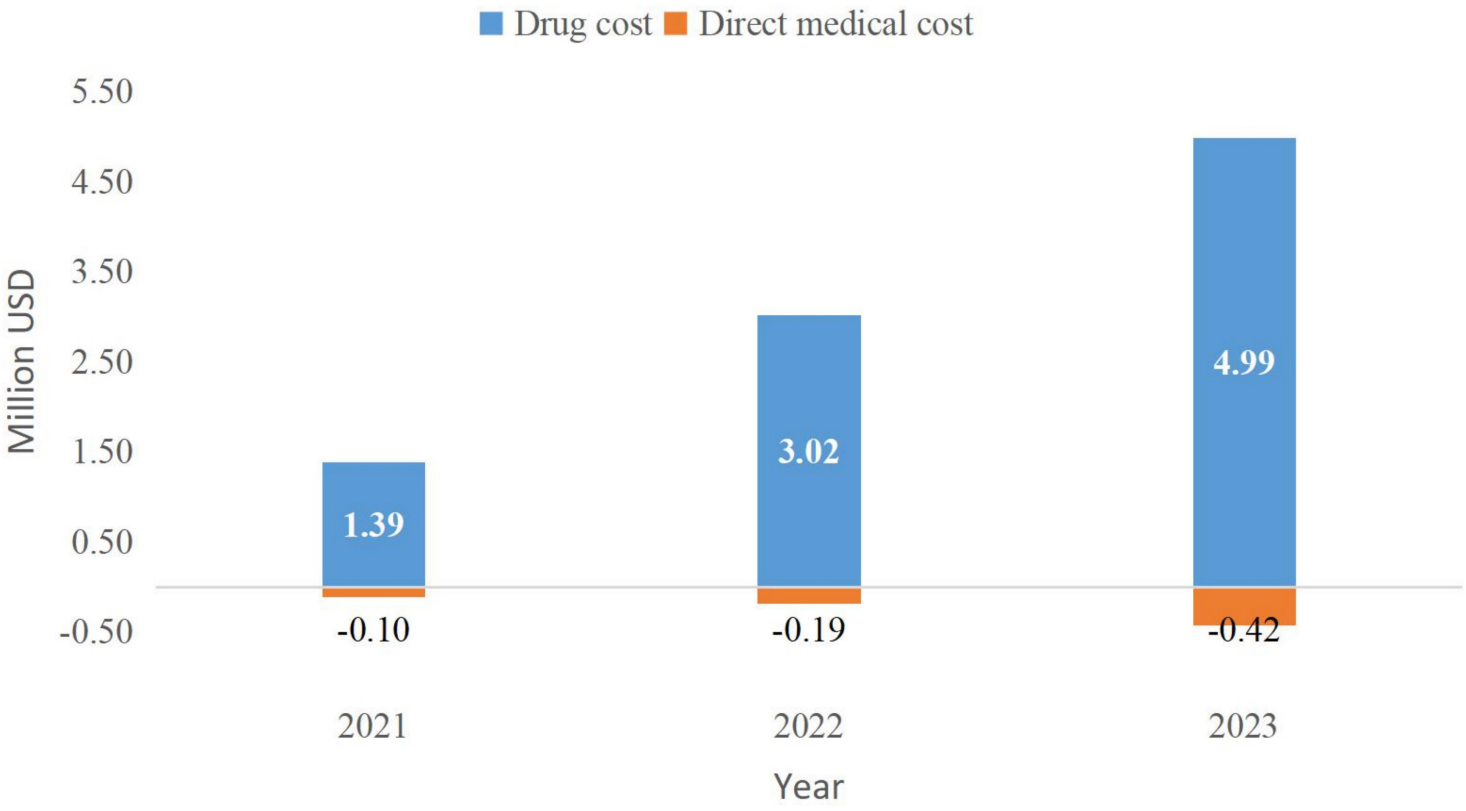
Incremental budget impact of the inclusion of PER in the NRDL (2021–2023). NRDL, National Reimburesement Drug List.

Before and after the inclusion of PER in the NRDL, the absolute BI over 2021–2023 will be $17.30, $27.78, and $39.08 million and $18.58, $30.61, and $43.65 million, respectively (Table 8). The incremental BI (including drug and medical service costs) over the 3 years will be $1.28, $2.83, and $4.56 million, respectively, accounting for 0.00037, 0.00082, and 0.00132% of the total expenditure of national medical insurance in that year (total medical insurance expenditure was acquired from medical insurance bureau reports. Among them, drug costs will increase by $1.39, $3.02, and $4.99 million, whereas medical service costs will be reduced by $0.10, $0.19, and $0.42 million (Figure 5). Medical insurance reimbursement costs per capita (including drug and medical service costs) will increase by $110.72, $151.59, and $169.14.

**Table 8:** Budget impact (Before and after the inclusion of PER in the NRDL), million USD.

**Costs**		**Before the inclusion of PER in the NRDL**				**After the inclusion of PER in the NRDL**			
	**2020**	**2021**	**2022**	**2023**	**3-Year total**	**2021**	**2022**	**2023**	**3-Year total**
**URRBMI&UEBMI**									
Total	10.61	17.30	27.78	39.08	94.77	18.58	30.61	43.65	103.45
Durg	5.60	9.33	15.16	21.25	51.34	10.72	18.18	26.23	60.73
Direct medical	5.01	7.96	12.62	17.83	43.43	7.86	12.43	17.41	42.71
**URRBMI**	7.14	11.83	19.17	27.15	65.29	12.74	21.12	30.42	71.42
**UEBMI**	3.47	5.46	8.61	11.93	29.48	5.84	9.49	13.23	32.03

*PER, perampanel; NRDL, National Reimburesement Drug List; URRBMI, Urban Rural Resident Basic Medical Insurance UEBMI, Urban Employee Basic Medical Insurance*.

#### Deterministic Sensitivity Analysis

**Figure 6:**
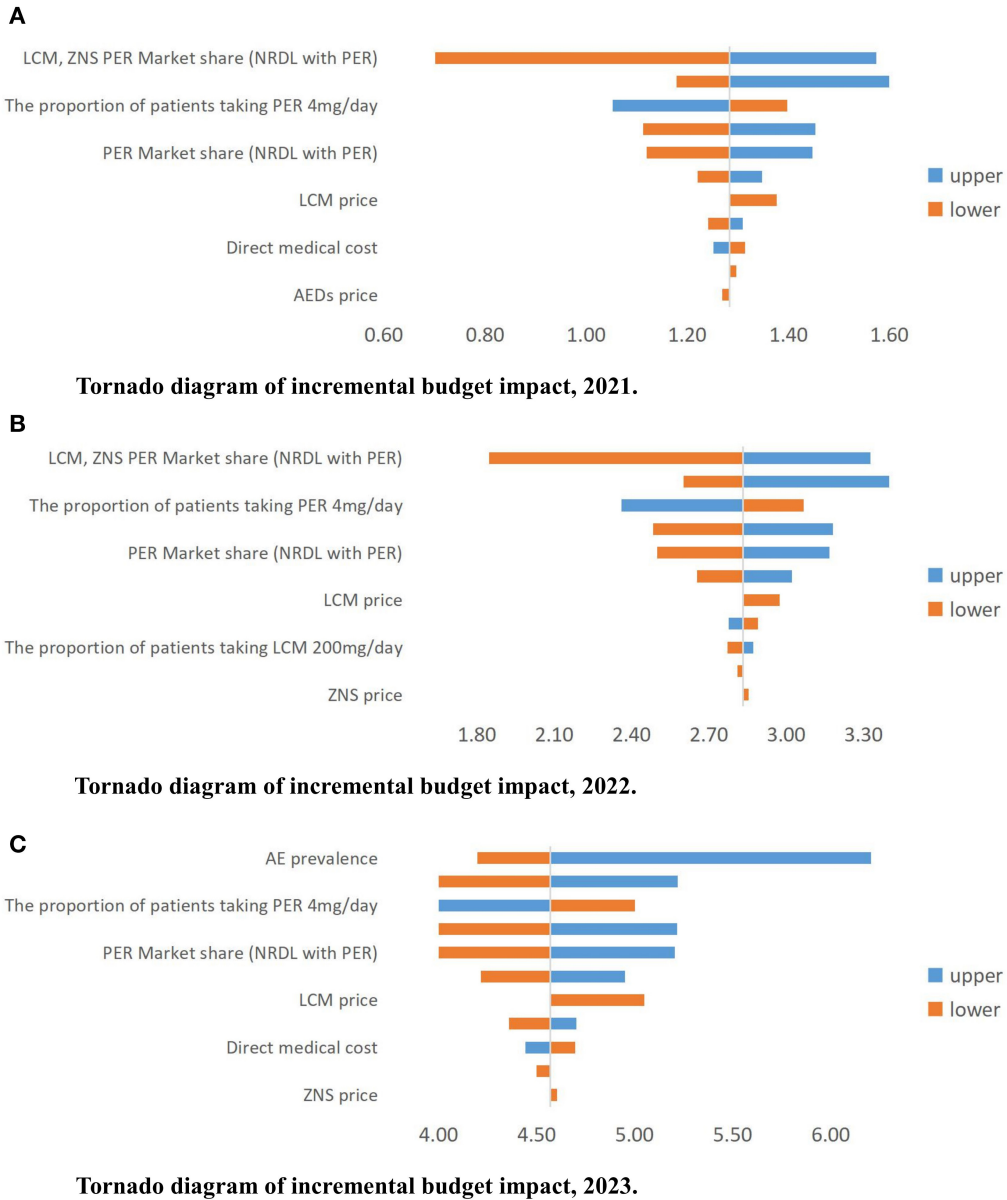
**(A–C)** Tornado diagram of incremental budget impact, 2021–2023 (million USD). AE, active epilepsy; PER, perampanel; LCM, lacosamide; ZNS, zonisamide; NRDL, National Reimburesement Drug List; AED, antiepileptic drug.

The BI results are relatively robust. The factors that have the greatest impact on the results are the market share after inclusion of PER in the NRDL, the prevalence of AE, and the distribution of patients using different daily doses of PER (4/8 mg). The price of PER also has a relatively large impact on the results (Figure 6).

## Discussion

This is the first study to calculate the value of PER as an add-on regimen to the treatment of patients with partial-onset seizures in China with respect to CE and medical insurance affordability. Currently, there are limited documents on the economic evaluation of PER. An economic evaluation based on the perspective of Spain showed that the ICER of PER as an adjuvant therapy to AEDs was €16,557/QALY over a 33-year study period; with the willingness-to-pay of €30,000/QALY, PER had an 89.3% probability of being the cost-effective option for primary generalized tonic-clonic epileptic seizures (18). Here, our study from the perspective of China's health system shows that using PER as an adjuvant therapy to AEDs has an dominate advantage over LCM in terms of CE. This result is also relatively stable within the one-two-three times GDP per capita of $10,838–$32,515. Moreover, the time horizon of our model is the patient's lifetime. Based on the BIA, this study is in agreement with the results of Tremebly et al. (19) for PER; that is, PER as an add-on brings about an increase in BI year by year while also resulting in a medical service cost savings. This result is mainly attributed to the improvement of health state after the use of PER, which in turn reduces the direct medical costs. In clinical trials 304, 305, and 306 with PER, 4.4% and 3.5% of the patients achieved a seizure-free state owing to an add-on therapy with PER at 4 or 8 mg/day (1.0% in the AEDs placebo group) (37), whereas in the clinical trial 335, the proportion of seizure-free patients in the Chinese population was even higher in the PER group (8.8 vs. 0%).

This study is novel in several ways. First, this study simulates the lifetime effectiveness and cost data of patients through a Markov model, with full consideration of long-term simulation and health state classification of epileptic patients. Epilepsy is a chronic disease that may be lifelong (1). It is therefore necessary to investigate the long-term health outcomes of epilepsy and distinguish the costs from different frequencies of epileptic seizures. In an economic evaluation of LCM compared with conventional therapy, Simoens and Bolin et al. adopted a decision tree model to simulate the outcomes of short-term therapy in epileptic patients without considering the seizure-free state (20, 21). Furthermore, no head-to-head studies on PER and LCM have been found, which is also one of the innovative points of this study.

Second, this study gives a comprehensive consideration to the daily dose of drugs. The WHO recommends the adult daily doses of PER and LCM to be 8 and 300 mg/day, respectively (24). Clinical trial 335 of PER involved three daily dose groups of 4, 8, and 12 mg, respectively (12). However, clinical trial EP0008 of LCM only consisted of two daily dose groups of 200 and 400 mg, respectively (16). According to KOL opinions, we finally determine to explore the CE of PER 8 mg/day vs. LCM 400 mg/day and PER 4 mg/day vs. LCM 200 mg/day. We did not include PER 12 mg/day in the analysis because this dose is mainly used in patients with refractory epilepsy as indicated by KOL. In the BIA, the distribution of patients using different doses is taken into account comprehensively in accordance with the actual use ratio in the clinic given by KOL, aiming to be more in line with the real-world situation.

Third, this study also takes into account multiple aspects of cost data to better reflect the current situation in China. All the drugs analyzed in this study are included in the latest NRDL (announced by medical insurance bureau at the end of December 2020). Considering the fairness of the control and the origin of efficacy data from original drugs, we obtain the price of LCM from the price of the original drug, whereas for the remaining AEDs, both original and generic drugs are taken into account to make the price close to the market. In China, the price of medical services is set by medical insurance bureaus of various provinces and cities, leading to the differences in medical service price across regions. Here, we select nine provinces and cities based on their geographical locations in East, Central, and West China, and then take the medians of their prices. In addition, considering that epileptic patients usually visit second- and third-level medical institutions, we have the prices of second- and third-level medical institutions weighted according to the proportion of the number of medical visits (32). In the BIA based on the perspective of medical insurance payers, we not only distinguish the difference in the reimbursement of various insurance categories (URRBMI and UEBMI), but also consider the difference in the reimbursement policies of outpatient, emergency, or inpatient, and Class A or Class B of drugs.

Our study is not without limitations. First, the lack of local data in China may introduce potential bias in the results. For example, because no data are available on the utility value and mortality risk caused by epilepsy in the Chinese population, the utility value (31) and mortality risk (30) of foreign population are used in this study. There are also no data on the long-term transition probability of epileptic patients in the Chinese population. We therefore use the transition of response rates in epileptic patients of a 5-year follow-up investigation conducted in the United Kingdom (27). Moreover, with regard to whether there will be a certain probability for epileptic patients with a response rate of <50% transitioning to increased seizures during actual drug use in the clinic, we take this situation as a scenario analysis for supplementation, because follow-up data are lacking for the Chinese population.

Further, we have to make certain assumptions because of the objective lack of some data. For example, LCM lacks the proportion of patients with increased seizures reported in clinical trials. We therefore assume that the proportion of patients whose seizures increased by <25% is equal to that of the PER group, whereas the proportion of patients whose seizures increased by ≥25% is based on another clinical trial (26). In the BIA, reports on the annual growth rate of the prevalence of AE is lacking. Thus, we select the annual growth rate of the lifetime prevalence of epilepsy reported in a meta-analysis in China (34) and perform a sensitivity analysis on this parameter. In addition, because no available clinical research reports on ZNS are found, we assume in the BIA that the proportion of AEDs and therapeutic efficacy of patients in the ZNS group are at the average levels of PER and LCM. We consult pharmacoeconomic experts and clinicians to deal with all the assumptions as much as possible.

Finally, some factors are not taken into account in this study, such as compliance and adverse reactions. Frequent administration of AED affects patient compliance. When AED is administered once a day, patient compliance is 87%; when AED is administered twice a day, patient compliance drops to 81% (PER once a day and LCM twice a day) (38). However, because the two drugs are both add-on therapy in this study and the daily administration frequency of AEDs also affects compliance, the measurement of compliance value is not included in the model. Previous research has reported that PER and LCM cause relatively mild adverse reactions in a short term (12, 16), whereas ZNS is currently a generic drug in the Chinese market which lacks reliable research reports. Long-term adverse reactions to PER and LCM need to be determined through further follow-up monitoring of the Chinese population.

## Conclusion

Our study demonstrates that PER is valuable as an add-on therapy for patients with partial-onset seizures in China. PER has a dominate advantage of CE compared with LCM (8 vs. 400 mg/day; 4 vs. 200 mg/day), and its incremental BI for medical insurance payers is relatively acceptable. In this study, the BIA as a supplement to CEA achieves a relatively comprehensive evaluation of the value of PER in the treatment of partial-onset seizures in China. China has not yet developed a value framework that integrates CE and BI proposed by foreign researchers (22). Nevertheless, evidence on the CE and affordability of drugs has received increasing attention in this country. The present study also provides a reference for stakeholders to judge the value of PER.

## Data Availability Statement

Publicly available datasets were analyzed in this study. This data can be found here: https://clinicaltrials.gov/ct2/show/NCT01618695?term=NCT01618695&draw=2&rank=1; https://clinicaltrials.gov/ct2/show/results/NCT01710657?term=NCT01710657&draw=2&rank=1.

## Ethics Statement

Ethical review and approval was not required for the study on human participants in accordance with the local legislation and institutional requirements. Written informed consent for participation was not required for this study in accordance with the national legislation and the institutional requirements.

## Author Contributions

DZ and XL were mainly responsible for research model building, data collection, and manuscript writing. WT was responsible for research design, model building, and manuscript review and modification. XK, WD, and YR contributed to the collection of model data and model building. HX provided beneficial help to the initial model building. JD confirmed the clinical and epidemiological inputs. All authors have contributed substantially to the study and approved the final submitted version of the manuscript.

## Conflict of Interest

The authors declare that the research was conducted in the absence of any commercial or financial relationships that could be construed as a potential conflict of interest.

## Abbreviations

CEA: cost-effectiveness analysis
BIA: budget impact analysis
ICER: incremental cost-effectiveness ratio
NRDL: National Reimbursement Drug List
AE: active epilepsy
AED: antiepileptic drug
PER: perampanel
LCM: lacosamide
LY: life year
QALY: quality-adjusted life years
DDD: defined daily dosages
KOL: Key Opinion Leader
DSA: deterministic sensitivity analysis
PSA: probabilistic sensitivity analysis
CEAC: cost-effectiveness acceptability curve
ZNS: zonisamide
URRBMI: Urban Rural Resident Basic Medical Insurance UEBMI, Urban Employee Basic Medical Insurance.
