# Supplementary material for: Value of Perampanel as Adjunctive Treatment for Partial-Onset Seizures in Epilepsy: Cost-Effectiveness and Budget Impact Analysis
Source: Front Public Health. 2021 Jul 6;9:670108. doi: 10.3389/fpubh.2021.670108 (PMC8290415; doi:10.3389/fpubh.2021.670108)
Supplement: Supplementary file 1 [file Data_Sheet_1.pdf]

Table S1 Patient characteristics at baseline in clinical trials

	PER 8mg/day (N=48)	LCM 400mg/day (N=133)	PER 4mg/day (N=43)	LCM 200mg/day (N=135)
Mean (±SD) age, years	27.35 (11.93)	30.30 (11.30)	27.73 (11.32)	32.20 (11.20)
Female sex, n (%)	44.90	38.40	46.67	47.10
Median (Range) seizure frequency per 28 d	8 (3-81)	8 (3-221)	7 (3-114)	9 (4-1118)
Median (Range) number of anti-epileptic drugs	2 (1-3)	2 (1-3)	2 (1-3)	2 (1-3)

PER, perampanel; LCM, lacosamide

Table S2 Response transition probabilities in four months, %

From/to	Maintenance therapy	Increase in seizure	<50% Response	50%-74% Response	75%-99% Response	Seizure free
**PER 8mg/day vs. LCM 400mg/day**						
Maintenance	100	0	0	0	0	0
Increase in seizure	100	0	0	0	0	0
＜50 Response	0	0	97.74	1.12	0.59	0.55
50-74 Response	0	0	3.18	96.00	0	0.81
75-99 Response	0	0	3.18	0	96.00	0.81
Seizure free	0	0	0.27	1.67	0.94	97.13
**PER 4mg/day vs. LCM 200mg/day**						
Maintenance	100	0	0	0	0	0
Increase in seizure	100	0	0	0	0	0
＜50 Response	0	0	97.74	1.00	0.70	0.55
50-74 Response	0	0	3.18	96.00	0	0.81
75-99 Response	0	0	3.18	0	96.00	0.81
Seizure free	0	0	0.27	1.49	1.04	97.19

PER, perampanel; LCM, lacosamide

Table S3 Medical service costs by health state

Resource	Unit Costs,$	Resource use, per 4 month			
		≥53 seizures/year	13-52 seizures/year	≤12 seizures/year	Seizure free
Outpatint visit	145.99	2.80	2.06	1.06	0.74
Emergency room visit	116.17	0.65	0.52	0.41	0.24
Hospitalization	154.16	0.42	0.40	0.36	0.23
Number of days per hospitalization	7.66	7.25	6.08	5.69	5.09
Total cost		570.77	440.76	273.17	180.33

Table S4 Health state distribution (defined by the frequency of seizures) before and after treatment with PER, LCM and ZNS.

No. of seizures/year	**PER**		**LCM**	
	Before	After	Before	After
⩾53	87.10	34.38	87.01	33.36
13-52	12.90	18.26	12.99	20.13
⩽12	0	3.10	0	3.62
Seizure free	0	7.47	0	6.02
mortality	0	36.78	0	36.87
Total	100	100	100	100

No. of seizures/year	**ZNS**		**AED maintenance**	
	Before	After	Before	After
⩾53	87.06	33.87	87.10	36.55
13-52	12.94	19.20	12.90	19.05
⩽12	0	3.36	0	2.39
Seizure free	0	6.75	0	3.47
mortality	0	36.82	0	38.54
Total	100	100	100	100

Above data showed the patients’ health states at the baseline and treated by different drugs after 5 years. Assuming baseline year is 2019. PER, perampanel; LCM, lacosamide; ZNS, zonisamide; AED, antiepileptic drug.

Table S5 Reimbursement policy of basic medical insurance

Type of reimbursement		Rate of reimbursement, %
URRBMI	Outpatient and emergency visits	43.42
	Hospitalization	59.70
UEBMI	Outpatient and emergency visits	59.40
	Hospitalization	75.60
Ratio of advance payment for class B drugs		10.00

a. The rate of reimbursement of URRBMI and UEBMI from reports of National healthcare security administration

b. Class A drugs includes valproic Acid, carbamazepine, oxcarbazepine, phenytoin, clonazepam, phenobarbital, estazolam, and Class B drugs includes lacomsamide, zonisamide, lamotrigine, levetiracetam, topiramate, gabapentin, nitrazepam, pregabalin. According to the reimbursement policy of basic medical insurance to be reimburse for Class A drugs, but patients need to first pay a certain proportion of fees and then enter the reimbursement of basic medical insurance for class B drugs.
